# Development and validation of a dynamic online nomogram for predicting acute kidney injury in cirrhotic patients upon ICU admission

**DOI:** 10.3389/fmed.2023.1055137

**Published:** 2023-01-27

**Authors:** Lu-Huai Feng, Yang Lu, Shuang Ren, Hengkai Liang, Lu Wei, Jianning Jiang

**Affiliations:** ^1^Department of Infectious Diseases, The First Affiliated Hospital of Guangxi Medical University, Nanning, Guangxi, China; ^2^Department of Comprehensive Internal Medicine, The Affiliated Tumor Hospital of Guangxi Medical University, Nanning, China

**Keywords:** acute kidney injury, prediction, model, intensive care unit, dynamic online nomogram

## Abstract

**Background:**

Acute kidney injury (AKI) is one of the most common and deadly complications among cirrhotic patients at intensive care unit (ICU) admission. We aimed to develop and validate a simple and clinically useful dynamic nomogram for predicting AKI in cirrhotic patients upon ICU admission.

**Methods:**

We analyzed the admission data of 4,375 patients with liver cirrhosis in ICU from 2008 to 2019 in the intensive care unit IV (MIMIC-IV) database. The eligible cirrhotic patients were non-randomly divided into derivation (*n* = 2,188) and validation (*n* = 2,187) cohorts at a ratio of 1:1, according to the order of admission. The least absolute shrinkage and selection operator regression model was used to identify independent predictors of AKI in the derivation cohort. A dynamic online nomogram was built using multivariate logistic regression analysis in the derivation cohort and then validated in the validation cohort. The C-index, calibration curve, and decision curve analysis were used to assess the nomogram’s discrimination, calibration, and clinical usefulness, respectively.

**Results:**

The incidence of AKI in 4,375 patients was 71.3%. Ascites, chronic kidney disease, shock, sepsis, diuretic drugs, hepatic encephalopathy, bacterial infections, vasoactive drugs, admission age, total bilirubin, and blood urea nitrogen were identified using the multivariate logistic regression analysis as significant predictors of AKI upon ICU admission. In the derivation cohort, the model showed good discrimination (C-index, 0.786; 95% CI, 0.765–0.806) and good calibration. The model in the validation cohort yielded good discrimination (C-index, 0.774; 95% CI, 0.753–0.795) and good calibration. Decision curve analysis demonstrated that the dynamic online nomogram was clinically useful.

**Conclusion:**

Our study presents a dynamic online nomogram that incorporates clinical predictors and can be conveniently used to facilitate the individualized prediction of AKI in cirrhotic patients upon ICU admission.

## Introduction

The global burden of cirrhosis is increasing. Almost two-thirds of the global cirrhosis mortality rate increased between 1990 and 2015, according to the Global Burden of Disease Study (1). Liver cirrhosis and its complications account for a significant portion of morbidity and mortality, especially for patients admitted to the ICU (2). One of the most common and deadly complications among hospitalized cirrhotic patients is acute kidney injury (AKI) (3, 4), which develops in about 20–50% of all hospitalized cirrhotic patients (4, 5) and is much higher (30–80%) in the ICU patients (6–9). In addition, AKI increases mortality seven-fold, with 50% of patients dying within one month (10). Therefore, preventive strategies for AKI are essential to reduce the mortality of liver cirrhosis and the economic burden of liver cirrhotic patients.

Currently, serum creatinine level and urinary volume are the most important clinical indicators of AKI diagnosis, according to the Kidney Disease Improving Global Outcomes (KDGIO) criteria (11). Some studies proposed that several molecules may serve as biomarkers for detecting kidney damage before serum creatinine increases, the most promising of which are cystatin C, kidney injury molecule-1, neutrophil gelatinase-associated lipocalin, and liver-type fatty acid binding protein, among others (12). However, current diagnostic biomarkers have significant diagnostic limitations (4, 9, 12). Thus, more effective tools for early prediction of AKI in cirrhotic patients should be further explored.

AKI and its impact on clinical outcomes for intensive care patients with cirrhosis have become more widely recognized in recent years. However, AKI in cirrhotic patients is usually multifactorial (9), and early diagnosis is still a challenge (13). Henceforth, we conducted this study with the aim of developing and validating a simple and clinically useful dynamic nomogram for predicting AKI in cirrhotic patients upon ICU admission.

## Patients and methods

### Data source

We followed the Transparent Reporting of Multivariable Prediction Models (TRIPOD) statement (14) to report the development and validation of this prediction model. A TRIPOD checklist of the present study is provided in the Supplementary materials. A retrospective case–control study was conducted using the data of the MIMIC-IV database, a large, publicly available database that includes de-identified health-related data of approximately 60,000 ICU admissions between 2008 and 2019, maintained by the Laboratory for Computational Physiology of the Massachusetts Institute of Technology (15). The records include details such as admission records, procedures, medications, International Classification of Diseases 9th edition (ICD-9) or ICD-10 diagnoses, demographics, laboratory tests, fluid balance, caregiver records, vital signs, and survival data. The establishment of MIMIC-IV (v 2.0) was approved by the institutional review boards of the Beth Israel Deaconess Medical Center (Boston, MA) and Massachusetts Institute of Technology (Cambridge, MA). Thus, this study was granted a waiver of informed consent. To get access to the MIMIC-IV database (v 2.0), one author (L-H F) completed the online training course of the National Institutes of Health (certification number 35897462).

### Participants

Patients diagnosed with cirrhosis identified by ICD-9 or ICD-10 codes and aged ≥18 years at the time of ICU admission were included in our study. Patients with AKI before being admitted to the ICU or without an ICU record were excluded from the study. In total, 18,032 patients with a diagnosis of cirrhosis were screened, and 4,375 adult patients were finally enrolled in the present study. According to the TRIPOD guidelines (14), all patients were non-randomly divided into derivation (*n* = 2,188) and validation (*n* = 2,187) cohorts at a ratio of 1:1, according to the chronological order of admission.

### Data extraction

Using Structured Query Language queries (PostgreSQL) tools (V.1.13.1), data were extracted from the MIMIC-IV database, including demographic characteristics, medical history, laboratory data, and final in-hospital diagnoses. Based on scientific knowledge, clinical importance, and predictors identified in previously published articles (13, 16–18), we extracted and analyzed the following data when AKI occurred:

- Demographic characteristics: admission age, gender, race, and mean blood pressure (mBp);
- Comorbidities: acute coronary syndrome (ACS), ascites, heart failure, malignant, chronic obstructive pulmonary disease (COPD), chronic kidney disease (CKD), diabetes mellitus, shock, sepsis, hepatic encephalopathy, hyponatremia, bacterial infections, and basic liver diseases;
- Medical history: use history of vasoactive drugs (phenylephrine, norepinephrine, epinephrine, dopamine, and dobutamine) and diuretic drugs (spironolactone, furosemide, and Aldactazide) according to patient medication records;
- Laboratory data: albumin (ALB), aspartate aminotransferase (AST), alanine aminotransferase (ALT), total bilirubin (TB), serum creatinine, blood urea nitrogen (BUN), international normalized ratio (INR), hemoglobin, platelets, potassium, sodium, lactate, prothrombin time, osmotic pressure, and glucose. Demographic characteristics were recorded during the first 24 h of ICU admission, and laboratory data were extracted at the time closest to the occurrence of AKI during hospitalization in the ICU admission. Comorbidities and the final diagnosis were identified using ICD-9 or ICD-10 codes.

### Outcome

The primary outcome in the present study was AKI during ICU stay defined according to the KDGIO criteria (11). Considering that the actual urine volume of patients would be affected after using diuretic drugs, we defined AKI only according to the change in serum creatinine value.

### Missing data handling

In the MIMIC-IV database, missing data is a common occurrence; however, excluding patients with incomplete data may lead to serious bias in the study. For missing data imputation, all variables used in the analyzes were taken into consideration. Less than 20% of missing values were found in all variables. Thus, all values were imputed as means for continuous variables with normal distributions and as medians for continuous variables with skewed distributions (19). Furthermore, none of the dichotomous variables used in our study had missing information.

### Statistical analyzes

The statistical analyzes were performed using SPSS version 26.0 (IBM Corp, Armonk, NY, United States) and R software (rms (20) and DynNom (21) in R version 4.2.1).^1^ All *p*-values were two-sided, and statistical significance was set at *p* < 0.05.

Categorical variables are expressed herein as percentages, while continuous variables are expressed herein as mean ± SD, median, or range, depending on their normality of distribution. Depending on the distribution of the variables, chi-square tests were used for categorical variables and *t*-tests or Wilcoxon rank sum tests for continuous variables between the two cohorts.

To enhance prediction accuracy and interpretability, least absolute shrinkage and selection operator (LASSO) regression analysis was used for selecting and regularizing variables (22). The features selected in the LASSO regression model in the derivation cohort were assessed using univariate logistic regression to evaluate variables for AKI (23). All variables with *p*-values <0.05 in the univariate logistic analyzes were further assessed by multivariable logistic regression using backward stepwise selection. Based on the fitted prediction model by multivariable logistic regression, we produced a clinical prediction nomogram and an interactive web-based AKI probability application with Shiny apps.

Calculations of discrimination and calibration were performed in both the derivation and validation cohorts to assess nomogram performance (24). The overall discrimination of the nomogram was ascertained in terms of the C-index, which ranges from 0.5 (no discrimination) to 1.0 (perfect prediction). The calibration of the nomogram was assessed by a visual calibration plot comparing the actual probability and predicted value of AKI occurrence. Furthermore, 1,000 bootstrap resamples were conducted for internal validation to assess the prediction accuracy of the nomogram. Additionally, a decision curve analysis (DCA) (25), which can calculate the net benefit of predictors and a model, was conducted to evaluate the clinical use of the nomogram.

## Results

### Patient characteristics

A total of 4,375 cirrhotic patients were enrolled, and their baseline characteristics are given in Table 1. The incidences of AKI were significantly different (*p* = 0.007) in the derivation (73.1%) and validation (69.5%) datasets. A total of 245 patients (11.2%) had stage 1 AKI, 570 patients (26.1%) had stage 2 AKI, and 785 patients (35.9%) had stage 3 AKI in the derivation cohort, while 255 patients (11.7%) had stage 1 AKI, 596 patients (27.3%) had stage 2 AKI, and 668 patients (30.5%) had stage 3 AKI in the validation cohort. Although the included patients were subgrouped according to the timing of admission, their clinical features and laboratory findings were comparable, indicating that they could be used both as derivation and validation data.

**Table 1:** Characteristics of patients in the derivation and validation cohorts.

Variable	Derivation cohort	Validation cohort	*p*-Value
Admission age, years	59 (52, 68)	60 (53, 68)	0.308
Race (*n*, %)			
White	1,486 (67.9%)	1,499 (68.5%)	0.001
Black	140 (6.4%)	195 (8.9%)	
Other	562 (25.7%)	493 (22.5%)	
Gender (*n*, %)			
Female	744 (34.0%)	794 (36.3%)	0.111
Male	1,444 (66.0%)	1,393 (63.7%)	
AKI, yes (*n*, %)	1,600 (73.1%)	1,519 (69.5%)	0.007
ACS, yes (*n*, %)	131 (6.0%)	124 (5.7%)	0.654
Ascites, yes (*n*, %)	1,174 (53.7%)	1,135 (51.9%)	0.244
CKD, yes (*n*, %)	476 (21.8%)	447 (20.4%)	0.286
COPD, yes (*n*, %)	100 (4.6%)	120 (5.5%)	0.165
Diabetes, yes (*n*, %)	722 (33.0%)	735 (33.6%)	0.669
Heart failure, yes (*n*, %)	408 (18.6%)	396 (18.1%)	0.645
Malignant, yes (*n*, %)	253 (11.6%)	240 (11.0%)	0.538
Shock, yes (*n*, %)	561 (25.6%)	595 (27.2%)	0.240
Sepsis, yes (*n*, %)	558 (25.5%)	567 (25.9%)	0.749
Diuretic drugs, yes (*n*, %)	1,318 (60.2%)	1,349 (61.7%)	0.327
Hepatic encephalopathy, yes (*n*, %)	277 (12.7%)	232 (10.6%)	0.034
Bacterial infections, yes (*n*, %)	617 (28.2%)	570 (26.1%)	0.112
Vasoactive drugs, yes (*n*, %)	564 (25.8%)	553 (25.3%)	0.709
Basic liver diseases			
Alcoholic	860 (39.3%)	841 (38.5%)	0.305
Biliary cirrhosis	37 (1.7%)	31 (1.4%)	
Viral hepatitis	417 (19.1%)	464 (21.2%)	
Alcoholic + viral	265 (12.1%)	237 (10.8%)	
Other	609 (27.8%)	614 (28.1%)	
Hyponatremia, yes (*n*, %)	569 (26.0%)	526 (24.1%)	0.136
ALB, g/dL	3.1 (2.7,3.5)	3.1 (2.7, 3.5)	0.024
ALT, IU/L	41.0 (21.0, 99.7)	39.0 (21.0,99.7)	0.484
AST, IU/L	77.0 (39.0, 175.7)	77.0 (39.0, 175.7)	0.477
TB, μmoI/L	51.3 (18.8, 94.1)	44.5 (18.8, 94.1)	0.144
Serum creatinine, μmoI/L	123.8 (70.7, 141.6)	114.9 (70.7, 141.6)	0.024
Blood urea nitrogen, mmol/L	10.7 (5.7, 15.0)	9.6 (5.7, 13.6)	0.169
mBp, mmHg	74 (68, 83)	74 (68, 83)	0.548
Hemoglobin, g/dL	9.5 (8.2, 10.5)	9.5 (8.2, 10.5)	0.403
Platelets, K/μL	109 (62, 133)	109 (64, 142)	0.220
INR	1.7 (1.3, 1.9)	1.6 (1.3, 1.8)	0.211
potassium, mmol/L	4.2 (3.8, 4.4)	4.1 (3.7, 4.4)	0.048
sodium, mmol/L	137 (134, 140)	137 (135, 140)	0.332
Lactate, mmol/L	2.1 (1.5, 2.6)	2.2 (1.5, 2.6)	0.125
PT, sec	17.9 (14.4, 20.7)	17.9 (14.5, 20.2)	0.526
Glucose, mmol/L	7.2 (5.7, 8.3)	7.2 (5.7, 8.3)	0.871
Osmotic pressure, mmol/L	303 (295, 308)	303 (295, 307)	0.562

AKI, acute kidney injury; ACS, acute coronary syndrome; COPD, chronic obstructive pulmonary disease; CKD, chronic kidney disease; ALB, albumin; AST, aspartate aminotransferase; ALT, alanine aminotransferase; TB, total bilirubin; mBp, mean blood pressure; INR, international normalized ratio; PT, prothrombin time.

### Predictor selection for the nomogram

**Figure 1:**
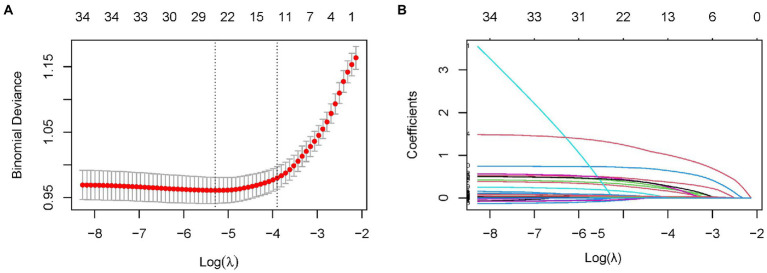
Predictor selection using the LASSO binary logistic regression model. **(A)** The tuning parameters (lambda) of the LASSO model were selected through 10-fold cross-validation using minimum criteria. A minimum criteria and a 1-SE criteria were used to draw the dotted vertical lines representing the optimal values. A lambda value of 0.016821, with log (lambda), 0.004167 was chosen (1-SE criteria) according to 10-fold cross-validation. **(B)** Profiling the 35 features by LASSO coefficients. Using log (lambda) sequences, a coefficient profile plot was generated. With 10-fold cross-validation, a vertical line was drawn at the value selected where optimal lambda resulted in 13 predictors with nonzero coefficients.

The LASSO regression analysis was used to reduce complexity by reducing 35 features to 11 potential predictors in the derivation cohort (Figures 1A,B). The predictors associated with AKI selected by LASSO regression are presented in Table 2 (lambda = 0.020260). Multivariable logistic regression analysis was used to further analyze the variables filtered through univariate logistic regression and LASSO analyzes.

**Table 2:** Univariate and multivariate logistic regression analyzes of variables relating to AKI in the derivation cohort.

	Univariate analysis		Multivariate analysis	
	OR (95% CI)	*p*-Value	OR (95% CI)	*p*-Value
Admission age	1.022 (1.014–1.030)	<0.001	1.017 (1.008–1.026)	<0.001
Ascites				
No	Reference	<0.001	Reference	0.009
Yes	1.938 (1.600–2.348)		1.345 (1.077–1.680)	
CKD				
No	Reference	<0.001	Reference	0.005
Yes	2.288 (1.751–2.989)		1.554 (1.142–2.115)	
Shock				
No	Reference	<0.001	Reference	0.012
Yes	4.587 (3.401–6.186)		1.639 (1.117–2.407)	
Sepsis				
No	Reference	<0.001	Reference	0.049
Yes	3.657 (2.763–4.840)		1.433 (1.001–2.050)	
Diuretic drugs				
No	Reference	<0.001	Reference	<0.001
Yes	2.714 (2.363–3.295)		2.185 (1.762–2.709)	
Hepatic encephalopathy				
No	Reference	<0.001	Reference	0.004
Yes	2.542 (1.780–3.630)		1.777 (1.207–2.615)	
Bacterial infections				
No	Reference	<0.001	Reference	0.001
Yes	2.330 (1.834–2.959)		1.601 (1.228–2.088)	
Vasoactive drugs				
No	Reference	<0.001	Reference	<0.001
Yes	7.089 (5.021–10.008)		3.708 (2.521–5.453)	
TB	1.075 (1.054–1.098)	<0.001	1.040 (1.017–1.063)	0.001
BNN	1.086 (1.069–1.103)	<0.001	1.046 (1.028–1.064)	<0.001

CKD, chronic kidney disease; ALT, alanine aminotransferase; TB, total bilirubin; BUN, blood urea nitrogen.

**Figure 2:**
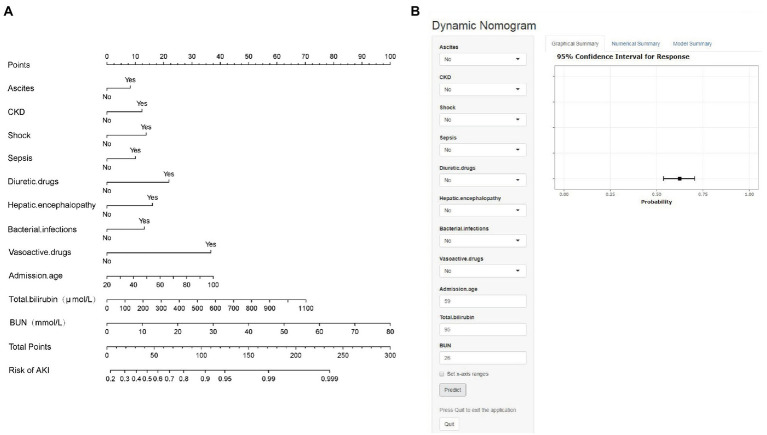
**(A)** Established nomogram for the prediction of AKI. **(B)** Online dynamic nomogram accessible at https://dynamicnomogram6666.shinyapps.io/dynnomapp/. The measurement units for total bilirubin and BUN in the model are μmol/L and mmol/L, and for total bilirubin, 1 mg/dL = 17.1umol/L, respectively.

The final multivariable logistic model identified 11 predictors (ascites, CKD, shock, sepsis, diuretic drugs, hepatic encephalopathy, bacterial infections, vasoactive drugs, admission age, total bilirubin, and BUN). The measurement units for total bilirubin and BUN in the model are μmol/L and mmol/L, and for total bilirubin, 1 mg/dL = 17.1umol/L, respectively. The nomogram (Figure 2A) represents the model that incorporates the independent predictors and is available online^2^ and presented as a screenshot in Figure 2B. To use the dynamic online nomogram, select “Yes” or “No” in the corresponding options, fill in the corresponding laboratory test results, and click “Predict” to determine the risk value of AKI of the patient.

### Performance of the nomogram in the derivation cohort

**Figure 3:**
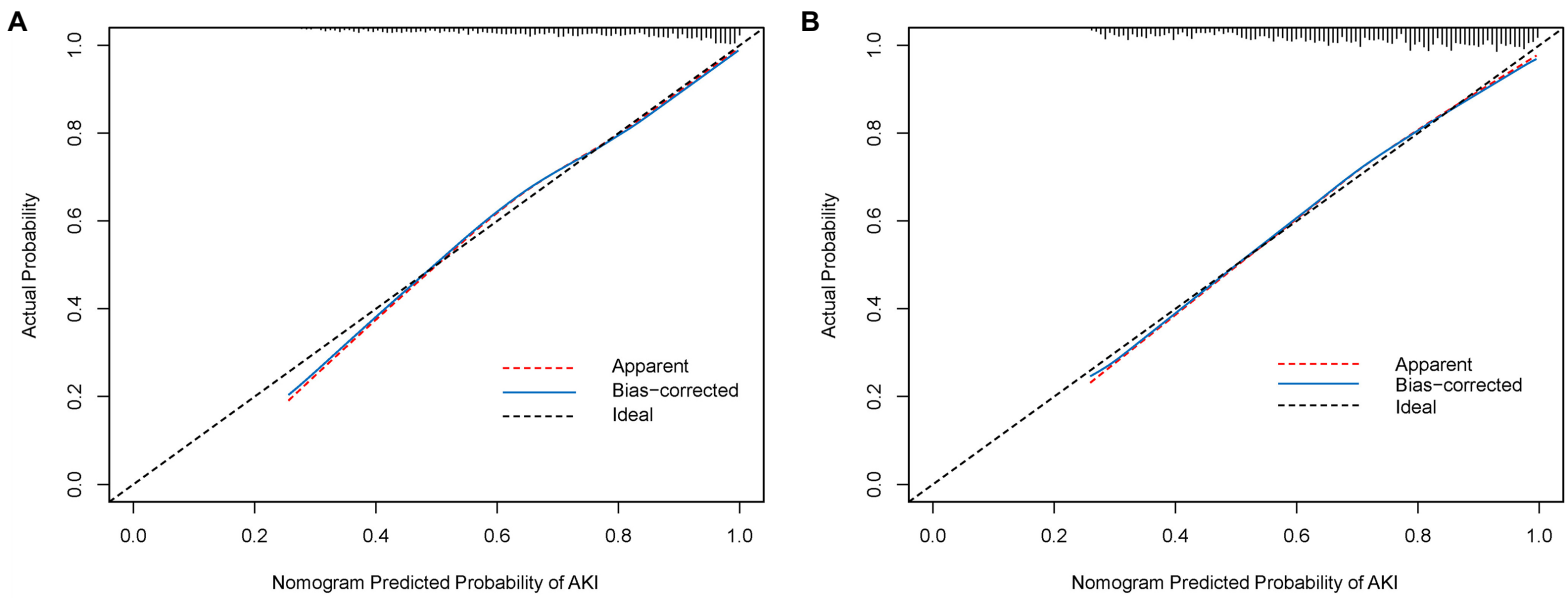
**(A)** Calibration curve of the nomogram in the derivation cohort. **(B)** Calibration curve of the nomogram in the validation cohort. The red dotted line represents the entire cohort, and the blue solid line is bias-corrected by bootstrapping (*B* = 1,000 repetitions), indicating observed nomogram performance.

As shown in Figure 3A, the calibration curve of the nomogram for the probability of AKI showed good agreement between the predicted and observed results in the derivation cohort. The Hosmer–Lemeshow test resulted in a non-significant statistic (*p* = 0.270), indicating that there was no over-fitting of the model. The C-index for the prediction nomogram was 0.786 (95% CI, 0.765–0.806) for the derivation cohort.

### External validation of the nomogram in the validation cohort

An external validation of the nomogram was conducted using bootstrap analyzes with 1,000 resamples. The calibration curve of the nomogram is plotted in Figure 3B. The probability of AKI in the validation cohort was well calibrated, with a mean absolute error of 0.01. The Hosmer–Lemeshow test resulted in a non-significant statistic (*p* = 0.768), which suggested that there was no departure from the perfect fit. The C-index of the nomogram for the prediction of AKI was 0.774 (95% CI, 0.753–0.795).

### Clinical use of the nomogram

**Figure 4:**
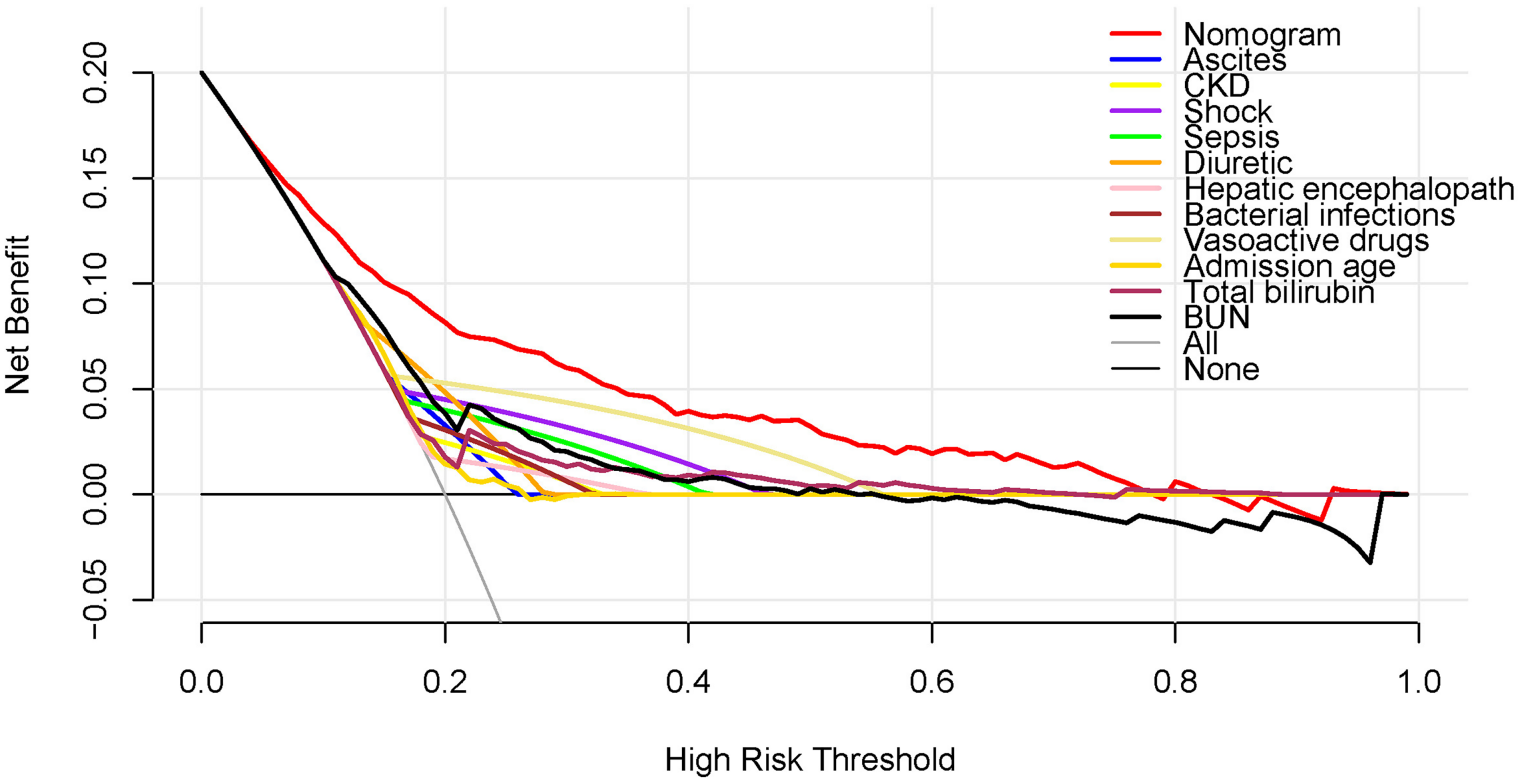
DCA of the nomogram.

Figure 4 shows the DCA results for the nomogram. The high-risk threshold probability is the probability of AKI predicted by the nomogram from which a clinician considers that the patient has an AKI risk and might benefit from the intervention. According to the decision curve, using the nomogram to predict AKI can offer a great deal of benefit if the threshold probability of a doctor is 10–80% and the predictive power of our nomogram is higher than that of a single predictor within this range.

## Discussion

In this study, we developed and validated a convenient and practical nomogram to predict the risk of AKI in ICU patients. We included 11 predictors, namely, ascites, CKD, shock, sepsis, diuretic drugs, hepatic encephalopathy, bacterial infections, vasoactive drugs, admission age, total bilirubin, and BUN, in our nomogram. The nomogram predicted AKI occurrence in cirrhotic patients upon ICU admission in a dynamic and easy-to-use online manner, responding to a move toward personalized medicine. Our model exhibited similar performance in the derivation and validation cohorts. Compared with considering all patients as non-AKI or considering all patients as AKI, the nomogram demonstrated good discrimination, calibration ability, and clinical usefulness with a net benefit greater than the net benefit of any single factor for a risk threshold between 10 and 80%.

Another particular strength of this study is that it considers a wide range of clinical variables previously reported to be associated with AKI in cirrhotic patients (4, 7, 26). Studies have shown that a history of ascites, age, spontaneous bacterial peritonitis, and the presence of sepsis/septic shock are risk factors for the development of AKI in cirrhosis (27, 28), which is congruous with our findings. In addition, researchers have endeavored to find markers of early renal insufficiency. Serum cystatin C, neutrophil gelatinase-associated lipocalin, and interleukin-18 are the most extensively studied biomarkers for AKI warning; however, the applicable cutoffs of these markers are not conclusive (29). The pathophysiology of AKI in liver cirrhosis is fairly complex, and heterogeneity is prevalent in the patient population of cirrhosis. Thus, a comprehensive evaluation tool that can provide valid risk prediction for individual patients must be developed. A few studies have developed predictive models of AKI in cirrhosis. To the best of our knowledge, the previous models are static. However, our nomogram is dynamic and can take input data at any time to quickly calculate AKI risk value. Few studies presented predictive models for AKI in cirrhosis. We believe that our dynamic online nomogram can help identify AKI in the early stages of cirrhosis and ensure a better short-term prognosis through timely intervention.

Cirrhotic patients should have an accurate AKI risk stratification since their prognosis may be heterogeneous. Instead of using diagnostic information from the KDGIO criteria, using data from large cohorts or population-based surveys. A dynamic online nomogram may provide a more individualized tool to provide AKI information to cirrhotic patients. Gameiro et al. (30) developed a risk score combining serum creatinine, MELD-Na, and neutrophil-to-lymphocyte ratio and demonstrated a strong discriminative ability to predict AKI in cirrhotic patients. However, in this study, we did not find that SCr had statistical significance in predicting AKI. It is problematic to include creatinine in the model, although serum creatinine level is the most important clinical indicator of AKI diagnosis. However, cirrhotic patients’ serum creatinine levels often overestimate their glomerular filtration rate by 50% because of reduced liver-produced creatinine, malnutrition, and reduced muscle mass, which can lead to a delay in diagnosing renal impairment. In addition, our study had the advantage of being carried out on a large number of patients (*n* = 4,375), and LASSO regression helped select the leading independent variables that predicted the AKI while considering significant correlations between dependent predictors.

It is essential to interpret an individual’s need for additional treatment or care when using the nomogram as the last argument. However, the performance of the risk-prediction model, discrimination, and calibration, may not reflect the clinical consequences of varying discriminatory levels or miscalibration levels (31). Therefore, we investigated whether dynamic online nomogram-assisted decisions would be useful in the early diagnosis of AKI in cirrhotic patients to justify the clinical usefulness of the system. To accomplish this, we performed a DCA, instead of multi-institutional prospective validation, which is largely impractical owing to heterogeneity in clinical data collection across institutions. With this novel approach, clinical outcomes can be analyzed on the basis of threshold probability, which yields the net benefit of treatment (32). According to the decision curve, if the threshold probability for a patient or doctor is 10–80%, using the dynamic online nomogram to predict AKI added more benefit than treating all patients or not treating any patients in the present study.

Despite its strengths, this study has a few limitations. First, this is a monocentric study in a single ICU, despite being done on a large cohort. Therefore, generalizing our results and dynamic online nomogram to other centers or countries is limited, and further research is required to validate this model in other places. Second, in LASSO regressions, two independent variables can be highly correlated, so the coefficients may drop arbitrarily, and ridge regressions may be better for this situation. However, the disadvantage of ridge regression is that it does not eliminate variables, so it is not recommended when creating a simple nomogram, which is meant to include as few variables as possible. Third, since the MIMIC database does not provide new biomarkers, such as cystatin C and neutral gelatin-associated lipocalin, we cannot further improve the predictive ability of this model. Fourth, as all such datasets, MIMIC-IV database suffers from the lack of quality control of input data. We relied only on accurate medical records of MIMIC-IV database to collect the data. In such an approach, there may be errors and problems associated with incomplete or missing data. Fifth, because this was a retrospective study, no bias could be avoided. However, we rigorously set the inclusion criteria so that both the control and case groups accurately reflected actual conditions.

## Conclusion

Our study presents a dynamic online nomogram that incorporates clinical risk factors and can be conveniently used to facilitate the individualized prediction of AKI in cirrhotic patients upon ICU admission. This tool may be helpful in selecting cirrhotic patients who benefit most from AKI prevention and treatment.

## Data availability statement

The raw data supporting the conclusions of this article will be made available by the authors, without undue reservation.

## Ethics statement

The establishment of MIMIC-IV(v2.0) was approved by the institutional review boards of the Beth Israel Deaconess Medical Center (Boston, MA) and Massachusetts Institute of Technology (Cambridge, MA); thus, this study was granted a waiver of informed consent. Written informed consent for participation was not required for this study in accordance with the national legislation and the institutional requirements.

## Author contributions

L-HF, YL, and JJ conceived the study. SR, HL, and LW carried out the research. L-HF and YL analyzed the data. L-HF, YL, and JJ wrote the paper. All authors have read and approved the final manuscript.

## Funding

This work was supported by the Guangxi Key Laboratory for the Prevention and Control of Viral Hepatitis (GXCDCKL202001), the Key Laboratory of High-Incidence-Tumor Prevention and Treatment (Guangxi Medical University), Ministry of Education (GKE-ZZ202107), the National Natural Science Foundation (81960115), the National Natural Science Foundation (82160123), and the National Natural Science Foundation (82260124).

## Conflict of interest

The authors declare that the research was conducted in the absence of any commercial or financial relationships that could be construed as a potential conflict of interest.

## Publisher’s note

All claims expressed in this article are solely those of the authors and do not necessarily represent those of their affiliated organizations, or those of the publisher, the editors and the reviewers. Any product that may be evaluated in this article, or claim that may be made by its manufacturer, is not guaranteed or endorsed by the publisher.

## Supplementary material

The Supplementary material for this article can be found online at: https://www.frontiersin.org/articles/10.3389/fmed.2023.1055137/full#supplementary-material

## Footnotes

^1^ https://www.r-project.org/

^2^ https://dynamicnomogram6666.shinyapps.io/dynnomapp/
